# Differences Between Flash Glucose Monitor and Fingerprick Measurements

**DOI:** 10.3390/bios8040093

**Published:** 2018-10-17

**Authors:** Odd Martin Staal, Heidi Marie Umbach Hansen, Sverre Christian Christiansen, Anders Lyngvi Fougner, Sven Magnus Carlsen, Øyvind Stavdahl

**Affiliations:** 1 Department of Engineering Cybernetics, Norwegian University of Science and Technology (NTNU), 7491 Trondheim, Norway; anders.fougner@ntnu.no (A.L.F.); oyvind.stavdahl@ntnu.no (Ø.S.); 2 Prediktor Medical, 1630 Gamle Fredrikstad, Norway; heidi@prediktor.no; 3 Department of Clinical and Molecular Medicine, NTNU, 7491 Trondheim, Norway; sverre.christiansen@ntnu.no (S.C.C.); sven.carlsen@ntnu.no (S.M.C.); 4 Department of Endocrinology, St. Olavs University Hospital, 7491 Trondheim, Norway

**Keywords:** blood glucose, measurement, error analysis, continuous glucose monitor, flash glucose monitor, self monitoring of blood glucose

## Abstract

Freestyle Libre (FL) is a factory calibrated Flash Glucose Monitor (FGM). We investigated Mean Absolute Relative Difference (MARD) between Self Monitoring of Blood Glucose (SMBG) and FL measurements in the first day of sensor wear in 39 subjects with Type 1 diabetes. The overall MARD was 12.3%, while the individual MARDs ranged from 4% to 25%. Five participants had a MARD ≥ 20%. We estimated bias and lag between the FL and SMBG measurements. The estimated biases range from −1.8 mmol/L to 1.4 mmol/L, and lags range from 2 min to 24 min. Bias is identified as a main cause of poor individual MARDs. The biases seem to persist in days 2–7 of sensor usage. All cases of MARD ≥ 20% in the first day are eliminated by bias correction, and overall MARD is reduced from 12.3% to 9.2%, indicating that adding support for voluntary user-supplied bias correction in the FL could improve its performance.

## 1. Introduction

People with diabetes need to control their blood glucose level to be as close as possible to the normal range, in order to avoid acute and chronic consequences of the disease. Continuous glucose monitoring (CGM) is an important tool for people with diabetes, primarily to detect potentially dangerous blood glucose levels and to assist in insulin bolusing. Secondarily, CGMs help patients with diabetes to understand the dynamics of their blood glucose levels, e.g. learning which foods give what glucose responses, which activities or situations trigger glucose fluctuation, or how their glucose level varies overnight. CGMs are so-called minimally invasive glucose measurement devices, meaning that they have a small electrochemical sensor inserted under the skin for the duration of the sensor wear, which at present is between seven and 14 days [1].

Due to the diffusion time of glucose from the capillaries to the subcutaneous interstitial fluid and diffusion across sensor membranes, CGM measurements are delayed compared to glucose measured in blood [2,3,4,5]. Consequently, CGM systems have a disadvantage compared to direct blood glucose measurements, and lag is a known issue with present CGM systems that users need to be aware of. However, recent CGM systems provide accurate results despite this lag, achieving Mean Absolute Relative Difference (MARD) of below 10% when comparing against blood glucose measurements [6].

A recent addition to the family of glucose monitoring devices is the Flash Glucose Monitor (FGM) [7], of which there is currently only one system on the market—Abbott’s Freestyle Libre (FL) [8]. The FL uses the same minimally invasive electrochemical sensing principle as conventional CGMs. A main difference between FGM and conventional CGM is in its usage; FL only provides a reading when the user scans the sensor using a hand held scanner device or a Near Field Communication (NFC) enabled smart phone. The current glucose level along with historic glucose data for the last 8 h is displayed on the scanner/smart phone. A consequence of this user-initiated data transfer between sensor and display unit is that the FL cannot provide alarm functionality, like CGMs do. Although the common usage pattern of FGM is different from CGM, in parts of this paper we treat the FGM data as if it is from a CGM, since the measurement principle is common between the systems, and our FGM data are frequently sampled.

CGMs have until recently required calibration against finger capillary blood measurements provided by Self Monitoring of Blood Glucose (SMBG) meters. Calibration is usually performed twice daily, and is intended to combat drift and minimize bias. However, the user supplied calibration values may also impair the overall accuracy of the system, e.g., if the user does not input the SMBG values correctly, the calibration is performed in periods of high glucose variation, or if the SMBG measurements are not correct. Calibration of CGMs is discussed in detail by Acciaroli et al. [9].

The FL is factory calibrated, meaning that the user does not need to calibrate it with SMBG measurements during the sensor wear. This is marketed as a profound improvement in the world of CGMs, and is likely responsible for a great deal of the popularity of the FL system, since the twice-daily SMBG calibration of most of its current CGM competitors is a burden to the users. However, the FL does not provide a means to bias-correct the measurements even if the user wants to. The FL has a built-in SMBG meter in the scanning device that would easily enable bias correction, but according to Bailey et al. [7], the built-in SMBG reader of the FL has no influence on the FGM readings. DexCom G6 is another calibration-free system that has recently been launched.

The introduction of calibration-free systems like FL eliminates the risk of failed calibration due to user error, but it comes with a price: it eliminates a mechanism that can reduce or ideally remove an inherent sensor bias due to either inaccurate factory calibration or some sensor-person interaction effect.

CGM/FGM data are useful for research purposes. For some uses it is important to identify the a model of the error in the datax. This has been done for conventional CGM systems by Facchinetti et al. [10]. The introduction of factory calibration means that a different model needs to be applied for systems like the FL. Characterization experiments like the one reported in this paper are needed to accomplish this. Being familiar with the characteristics of FGM signals and any limitations or challenges related to factory calibration is also important to patients and health care professionals.

In this paper we investigate the accuracy of FL measurements compared to SMBG measurements, and we investigate in detail the characteristics of the errors in the FL data, focusing on biases and lags in the FL glucose estimates. This has previously been requested by other researchers [11].

The current paper expands upon work presented orally at the Advanced Technologies & Treatments for Diabetes (ATTD) conference in Vienna, February 2018.

## 2. Method

### 2.1. Data Collection

In a study of 39 individuals with Type 1 diabetes, simultaneous SMBG (Freestyle Freedom Lite, Abbott) and FGM (Freestyle Libre, Abbott) measurements were taken every 10 min. These sessions lasted from 2.5–6 h and were performed in a research ward within 24 h of FL sensor insertion and activation.

**Figure 1:**
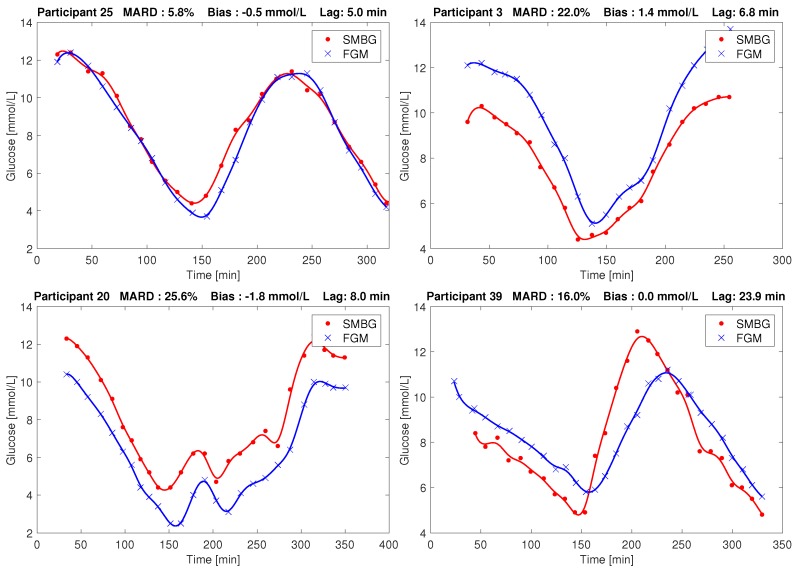
Examples of Flash Glucose Monitor (FGM) and Self Monitoring of Blood Glucose (SMBG) recordings in our study. **Top left:** Low Mean Absolute Relative Difference (MARD). **Top right:** Large positive bias. **Bottom left:** Large negative bias. **Bottom right:** Large lag. Points are measurements, and the line between points is the result of smoothing, as described by Staal et al. [12].

In these sessions the participants were non-fasting and used their regular insulin regime and sugary drinks or meals to manipulate their glucose level. The collected glucose responses typically had three flanks (i.e., up/down/up, or down/up/down) with approx. 5 mmol/L as the lower turning point and approx. 10 mmol/L as the upper turning point, respectively. The overall range of glucose in our dataset as measured by SMBG was 3 mmol/L to 26 mmol/L. Four representative examples of the SMBG and FGM data resulting from these sessions are shown in Figure 1.

After the frequent-sampling session, the participants went on to use the sensor in their everyday environment for days 2–7 after insertion, performing sporadic SMBG measurements and scanning the FL at least every 8 hours. Of the 39 sensors, 11 were dislodged before the 7 days had passed. Data from these sensors were included in the analysis, implying that for these participants, the data from days 2–7 are incomplete.

All participants signed consent forms prior to the study. The study was approved by the Regional Ethics Committee (REK Midt 2016/1172). The participant demographics is given in Table 1.

The paired FL and SMBG measurements are included in the supplementary data. The demographics data are not made publicly available, since they may enable identification of the study participants.

### 2.2. Data Analysis

Looking at the initial session (day 1) data, the MARD between corresponding FL scanned values ($y_{FGM}$) and SMBG data points ($y_{SMBG}$) was computed, both on an overall level and on a per individual level ($MARD_{p}$).

(1) $$MARD=\frac{1}{N_{all}}\sum_{i=1}^{N_{all}}\frac{|y_{i_{FGM}}-y_{i_{SMBG}}|}{y_{i_{SMBG}}}$$

(2) $$MARD_{p}=\frac{1}{N_{p}}\sum_{i=i_{p}}^{i_{p}+N_{p}}\frac{|y_{i_{FGM}}-y_{i_{SMBG}}|}{y_{i_{SMBG}}}$$

here $N_{all}$ is the overall number of paired points in our study. $i_{p}$ is the first measurement from participant *p* and $N_{p}$ are the number of paired points for participant *p*.

A Parkes/Consensus error grid (PEG) analysis [13] was performed on an overall level, using all paired points.

The data from all runs were plotted to investigate the characteristics and reason for high MARDs, and it was seen that bias and lag effects were present in the data. Therefore, an estimator based on a Rauch-Tung-Striebel Kalman smoother [12,14,15] was implemented to estimate the bias and lag from the data, using the following dynamic model of the FGM measurements:

(3) $$\begin{array}{cc} {\dot{G}}_{i} & =\frac{1}{\tau_{i}}\left(G_{p}-G_{i}\right)+v\left(t\right) \end{array}$$

(4) $$\begin{array}{cc} y_{FGM,k} & =G_{i,k}+b_{FGM}+w_{k} \end{array}$$

Here, $G_{p}$ is plasma glucose, $G_{i}$ is interstitial fluid glucose, and $\tau_{i}$ is a time constant governing the diffusion between these compartments. This time constant also models any diffusion dynamics across the FGM sensor membranes. The process noise is modeled by v(t). Furthermore, $y_{FGM,k}$ is the FGM measurement at time step k, $b_{FGM}$ is a bias constant, and $w_{k}$ is the measurement noise process. The parameters $b_{FGM}$ and $\tau_{i}$ were estimated per individual data set using the Kalman smoother and the above model, using the same noise modeling as Staal et al. [12]. The word “lag” is sometimes interpreted as a pure time delay, or a combination of a pure time delay and a time constant [16], but in this paper we use lag as a synonym for the time constant.

We investigated the effect on MARD of correcting the day 1 data for only bias, only lag, and both. The bias correction was done simply by subtracting the estimated bias $b_{FGM}$ per participant from all measurements $y_{i_{FGM}}$ from that participant, producing $y_{FGM,Bcorr}$. The lag correction is more complicated, and can be done in different ways [17,18]. We used Equations (3) and (4) in a Kalman smoother that use the participant lag, bias and FGM data to produce lag- and bias-corrected FGM measurements, $y_{FGM,BLcorr}$. By not providing the bias to the smoother we can produce an only lag-corrected signal, $y_{FGM,Lcorr}$, to investigate the effect on MARD of only correcting for lag. By providing neither bias nor lag information, we can produce an uncorrected signal $y_{FGM,smoothed}$ that has been subjected to all the processing of our method but has not really corrected for anything. This latter signal was used to get an idea of how much MARD is influenced by the smoothing introduced by our method. Both Kalman smoothers described above for parameter estimation and state estimation are working in fixed interval mode, i.e., they use all data in the data set, thus this is an offline method. A detailed description of the state estimation Kalman filter is provided in Appendix A.

**Figure 2:**
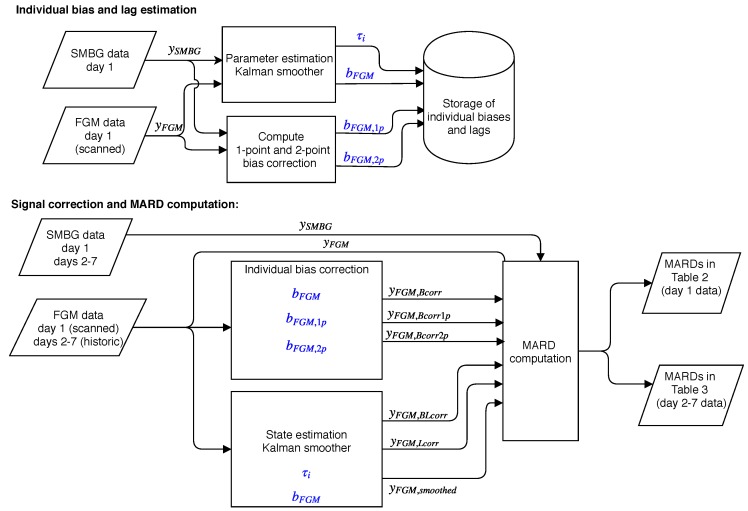
An overview of the signal processing. Items in blue are individual parameters estimated from day 1 data, and used to correct both day 1 and days 2–7 data for that individual. The parameters and signals shown are described in the main text, Section 2.2.

The bias and lag estimates computed by the Kalman smoother are accurate because of the many points used. Basing a real-time bias estimation on this method is not practical due to the many SMBG measurements required. In a practical bias calibration, only one or two data points per day should be used, as in normal CGM calibration regimes. We therefore also computed a 1-point bias correction, finding a bias $b_{FGM,1p}$ based on the error in the first paired data point from each day 1 session. Correcting for this bias produces a signal $y_{FGM,Bcorr1p}$. We also computed a 2-point bias $b_{FGM,2p}$ based on the mean of the errors in the first and last paired point from each session, producing the signal $y_{FGM,Bcorr2p}$. The different biases were used to correct data from day 1 and days 2–7. A block diagram providing an overview of the method we employed is shown in Figure 2.

To investigate whether the biases seen in the first day of use persisted throughout the use of the sensor, we performed an analysis of the data from days 2–7. Participants were grouped based on the bias they had in the first day. Individuals experiencing more than 1 mmol/L bias in day 1 were assigned to the positive bias group. Those that had less than −1 mmol/L bias were assigned to the negative bias group. The remaining participants were assigned to the unbiased group. The data were plotted based on this grouping, and the mean and standard deviation was computed in each group, for both day 1 and day 2–7 data. A *t*-test was performed to determine if the grouped means in days 2–7 were significantly different from the unbiased group. We also used a t-test to determine if the group means changed from day 1 to days 2–7. A similar analysis for the lag was not possible, due to the sparsity of data in days 2–7.

Finally, we checked if participant characteristics were associated with the observed biases and lags. We investigated BMI, height, weight, age, sex, duration of diabetes, use of blood pressure medication, use of any other medication and whether or not the sensor fell off during days 2–7. For binary variables (sex, medication use, sensor fall-off) we used a *t*-test at *p* = 0.05 to test if there was a difference in the mean between groups. For continuous variables we performed a linear fit and estimated the 95% confidence interval (CI) of the correlation coefficient. If this CI spanned 0, the correlation was considered insignificant.

As reported by Pleus et al. [19], the FL generates two time series of glucose estimates that are available in its export file. Scanned glucose is the instantaneous glucose the sensor estimates at the time when the sensor is scanned. Historic glucose is generated by the sensor every 15 min independently of the scanning. Up to 8 h of historic data are stored in the sensor and transferred to the display unit as part of a scan. We used the scanned glucose values in the analysis of the high-frequency sampling session (day 1), since SMBG measurements and FGM scans were performed simultaneously in this session. In the analysis of days 2–7, we used historic glucose values since there were too few occurrences of concurrent FL scans and SMBG measurements during the normal use in an everyday environment. The historic glucose values were interpolated as described by Staal et al. [12] to enable matching with the SMBG measurements.

## 3. Results

### 3.1. Overall MARD Analysis in Day 1 Data

**Figure 3:**
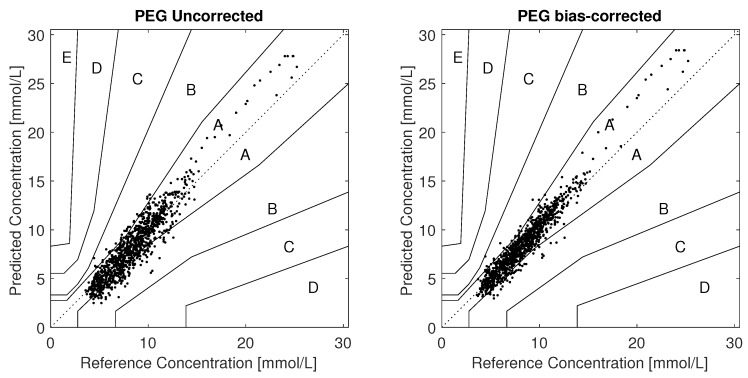
Parkes/Consensus Error Grid plots. **Left:** Uncorrected **Right:** Bias corrected Bias correction per individual makes 91.2% of the paired points lie in zone A.

There were 1053 paired measurements from the frequent-sampling sessions in day 1, having an overall MARD of 12.3%. A Parkes/Consensus error grid (PEG) analysis found all measurements to be within zones A+B, with 81.7% in zone A. The PEG plot is shown in Figure 3.

### 3.2. Day 1 Individual Participant MARD, Bias and Lag Analysis

**Figure 4:**
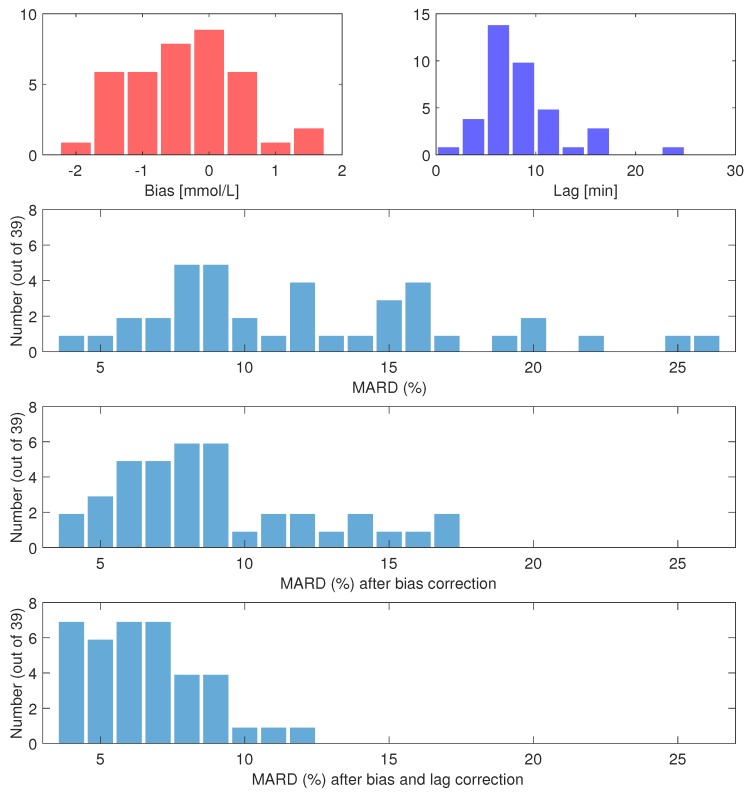
Histograms from day 1. **Upper left:** Estimated individual biases, $b_{FGM}$. **Upper right:** Estimated individual time constants, $\tau_{i}$. **Second row:** individual MARDs, uncorrected. **Third row:** individual MARDs, bias corrected. **Bottom row:** individual MARDs, bias and lag corrected.

There were on average 27 paired measurements per individual. Individual participant MARDs varied between 4.0% and 25.5%. A total of 18 participants had MARD <10%, however five had MARD ≥ 20%. Biases varied between −1.8 mmol/L, and +1.4 mmol/L, with a mean of −0.4 mmol/L. Time constants ranged from 2 min to 24 min, with a mean of 9 min. Histograms of individual MARDs, biases and lags on day 1 are shown in Figure 4. The most extreme cases of bias and lag are shown as time series in Figure 1. Other effects besides bias and lag are seen in some sets, e.g., overestimation in periods of high glucose (data not shown).

**Figure 5:**
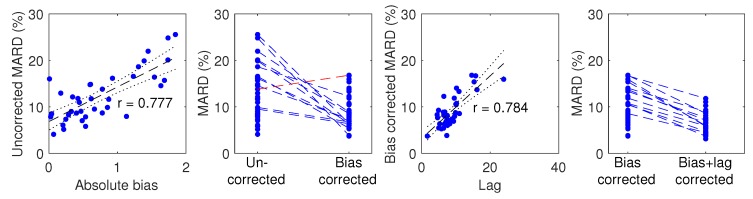
Effect of bias and lag correction. **Left:** Uncorrected MARD plotted against absolute bias. **Middle left:** MARD changes as a result of bias correction with individual participant tracing. Points belonging to the 12 participants whose MARD changed by more than 3 percentage points are connected by a dotted line (blue when MARD is reduced; red when MARD is increased). **Middle right:** Bias corrected MARD plotted against lag. **Right:** MARD changes as a result of lag correction. Regression lines are plotted in dashed black and 95% CIs in dotted black.

If the data are corrected for the biases found, the overall MARD on day 1 falls significantly (*p* < $10^{-6}$) from 12.3% to 9.2%. The participant with the largest MARD (25.5%) got a MARD of 6.4% with bias correction. The maximal individual MARD after bias correction is 17%. Of the 39 participants, 27 got a MARD below 10% after bias correction. For nine participants, the bias correction led to an increase in MARD, with one participant getting an increase of more than 3 percentage points. The MARD after bias correction is well correlated with the estimated lag; see Figure 5.

Compensating only for lag gave an overall MARD of 11.7%, while compensating for both bias and lag resulted in an overall MARD of 6.6%. The overall MARD, MAD and PEG zone A and A+B of uncorrected and corrected FGM data are listed in Table 2. Similar analyses for days 2–7 are given in Table A1 in Appendix B.

### 3.3. Persistence of Biases through Days 2–7

**Figure 6:**
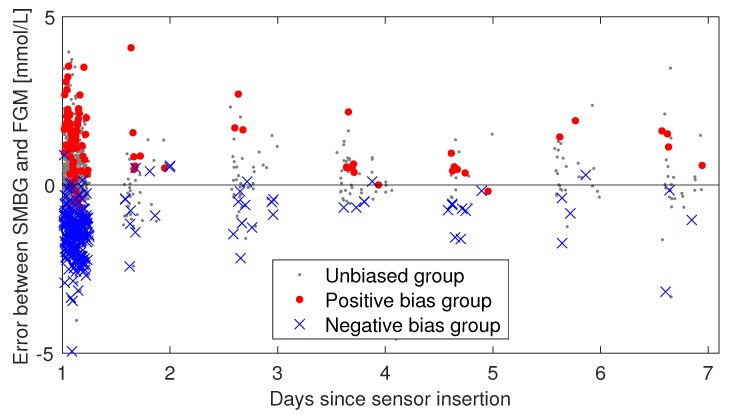
Persistence of bias in days 2–7. The individual sensor errors (SMBG–FGM) are plotted against time and colored according to what bias group the participant was in at day 1. If the bias observed in day 1 is not persistent, one would expect the blue and red data points to start mixing as time progresses. The day 1 session is included in this plot, but the analysis of bias persistence considered data from days 2–7 separately from day 1 data.

There were 356 data point pairs from days 2–7, these were plotted using the negative, positive and unbiased grouping based on day 1 biases, as described in Section 2.2. The plot is shown in Figure 6. The results of the grouped analysis of these data are given in Table 3. The difference of the mean between paired points from days 2–7 from each biased group compared to the paired points from the unbiased group is significant, judging by a *t*-test (*p* < $10^{-6}$). Within each group, the difference of the mean between paired points in day 1 and day 2–7 changes insignificantly, except for the negative bias group, which has a significant change (*p* < $10^{-6}$) moving towards zero.

### 3.4. Participant Factors vs. Bias and Llag

In the 18 comparisons we did to investigate correlation between bias, lag and the factors listed in Section 2.2, we found only one correlation with p<0.05. This was between lag and age (*p* = 0.046). Applying a Bonferroni correction, the p-level needed to achieve significance is 0.0027, thus this lag-age correlation is also considered insignificant.

## 4. Discussion

Several studies have reported an overall performance of the FL comparable to that of other state-of-the-art CGM systems [7,20,21,22,23,24,25,26,27,28,29]. Our main results are in line with previous observations as we find an overall MARD of 12.3% and half of the participants having a MARD at or below 10%. However, five of our 39 participants experienced a MARD at or above 20%. Several of the previous FL performance studies also report individual MARDs, where some are as large as we observed in our study [7,22,27]. The studies by Ólafsdóttir et al. [21] and Alsaffar et al. [24] report a bias in the measurements, but on an overall level only. To the best of our knowledge, the present study is the first to report on individual bias and lag issues as reasons for the high variation in individual MARDs in FL.

We observe that a bias is present in several of the the worst-performing sensors, and that bias correction significantly improves MARDs, both overall and on the individual level for most participants, see Figure 5. We can speculate that either the factory calibration is not accurate for these sensors, or that these biases arise from person-sensor interaction effects that are not predictable from any of the participant factors we investigated.

For some participants, bias correction increased MARD slightly, e.g., the participant shown in red in Figure 5. There are several reasons why this may happen. Firstly, since we only correct the bias, and not the lag, the MARD may go up. Secondly, the bias is found in a way that does not try to optimize MARD. Thirdly, since only bias and lag are modeled in our method, the bias estimate may be inaccurate if other errors are present in the data. We do see effects in some of the data sets that are not explainable by only bias and lag. For instance, we saw some cases of overestimation of high glucose values that looks like a gain issue. The data from participant 3 shown in the upper right of Figure 1 shows this tendency. An increase in MARD after bias correction can happen in datasets where effects other than bias and lag are present, or in datasets where the lag is large. The former could lead to inaccurate bias/lag estimation by the Kalman smoother, because the model it uses (Equations (3) and (4)) accounts for no other effects than bias and lag. The latter, large lag, may be a cause of increased MARDs after bias correction, since the MARD computation penalizes deviations from low reference values more than the same deviation from high reference values. The bias correction may well lead to less alignment of the low glucose values to give better alignment of the high glucose values, which will increase MARD. In our investigation of participant factors we found only one barely significant correlation (p=0.046) between age and lag, shown in Figure 5. We assume that this is a spurious correlation, firstly because we did enough comparisons to make it likely that one of them shows significance at p = 0.05 even if the underlying data are truly uncorrelated. Secondly, the negative correlation goes against our intuition about how tissue develops with age; if anything, we would expect higher age to give more delay between blood and interstitial fluid, not less.

Our study was not designed to resolve the matter of whether the biases are linked to the sensor or the individual. This is an obvious follow up question to the present study. Answering this question would require a study with at least two FL sensors per person, for instance as in the study performed by Freckmann et al. [29], where each of the 20 participants wore two FL sensors and two DexCom G5 sensors (DG5), and SMBG was measured every hour during three clinic visits. A larger between-sensor discrepancy was seen in FL than in DG5, as measured by Precision Absolute Relative Difference (PARD), and it was seen that four of 20 participants had a PARD ≥ 15. This is supported by the biases we observed in our study, and suggests that the biases might follow the sensor rather than the individual. Further, we can speculate that since DG5 is a conventional CGM which requires calibration SMBG measurements twice daily, the increased PARD of DG5 over FL could be caused by factory calibration issues in the FL. In a study performed by other researchers in our group [30], it was seen that the in vitro responses of four different sensors had a tendency to be offset from each other. This result also points at the sensor as the source of the bias rather than some sensor/participant interaction effect.

Insights into the factory calibration process of FL is provided by Hoss and Budiman [31]. The FL factory calibration is achieved through low sensor-to-sensor variability within a sensor lot, and performing in vitro tests of a sample of sensors within a lot to produce a factory calibration valid for all sensors of that lot. The authors state that “The factory calibration process is based on the assumption that the in vitro sensor sensitivity predicts the in vivo sensor response”. While this assumption may be true on an overall level where data from 10 or more sensors and individuals are pooled and averaged, it allows having significant errors on the individual level. This may be what we are observing.

A limitation of our study is that our data set did not include blood glucose measurements using lab glucose analyzers, e.g., YSI 2300 Stat Plus. Had such data been recorded we would have been able to eliminate SMBG measurement bias as a possible cause of the FGM vs SMBG biases. Since SMBG measurement errors have been reported to not be correlated in time [32], and SMBG measurements are not reported to exhibit biases of the magnitudes we observed [33,34], we are inclined to believe that the bias is a problem with the FL measurements, not the SMBG measurements.

Figure 6 and Table 3 indicate that the individual biases persist in days 2–7, however, the negative bias group seems to be moving towards zero. More frequent data sampling in days 2–7 from more participants would be needed to confirm that this is the case. To confidently answer the question of how the bias develops throughout a sensor session, bias must be accurately determined per day, requiring simultaneous estimation of the lag, like we did for day 1. To accomplish this we would have needed several frequent sampling sessions throughout the 14-day sensor lifetime. An alternative is to minimize the influence of lag by making sure that SMBG measurements used for calibration are taken in periods of low glycemic variation, as is the recommended practice in calibration of CGMs. Our data set contains too few such periods.

**Figure A2:**
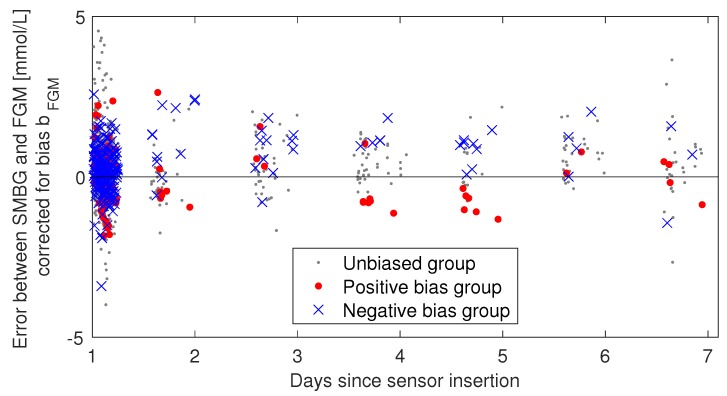
Same plot as in Figure 6, but with individual bias correction ($b_{FGM}$) applied. Using biases found from day 1 to correct day 2–7 data seems to give over-correction, at least in the negative bias group.

The performance of the bias correction on day 2–7 data indicate no significant improvement from any of the bias correction methods we applied, see Table A1 in Appendix B. The data from day 1 in our study is unsuitable for finding an accurate bias using only 1- or 2-point calibration, due to the lack of periods of low glycemic variation. This could be part of the reason why these corrections failed to improve MARD. However, the “best possible” multipoint bias estimated using a Kalman smoother also failed to improve MARD in days 2–7. This could be an indication that the bias changes over time, which seems to be the case for the negative bias group of participants (see Table 3). If so, a bias correction based on day 1 data would lead to over-correction in days 2–7, which seems to be the case at least for the negative bias group, see Figure A2 in Appendix B. The non-improving MARDs could also be caused by lag in the data. However, when we tried to also correct for the lag observed in day 1, this did not improve MARDs in days 2–7 (see Table A1), indicating that what is measured in day 1 is insufficient to correct the situation in days 2–7. Another explanation could lie in the difference between “scanned” and “historic” glucose values in the Freestyle Libre, as we based the bias estimate on scanned values from day 1 but correct historic values in days 2–7. Further research is needed to answer these questions, using more frequent SMBG sampling of data in days 2–7.

Assuming that the bias and lag observed in day 1 stays constant throughout the sensor lifetime seems like an invalid assumption to make. There are several physiological and technical reasons why both lag and bias may vary during the sensor lifetime, some of which are:

1. The insertion of the sensor into the interstitial fluid introduces local trauma to and/or minor bleeds in the tissue around the sensor, altering the glucose flow, thus making the sensor less accurate in the time immediately after insertion [1].
2. Biofouling of the sensor contributes to making sensor characteristic changes over the wear time likely.
3. On the technical side, the electrochemical sensor may suffer from non-physiological drift in the initial period of sensor wear [9].
4. Effects like Pressure Induced Sensitivity Attenuation (PISA) [35] may be present in the FGM data.

In addition, SMBG measurement outliers can occur, which will be difficult to detect and compensate for when there are only a few SMBG data points per person per day. Increasing the number of participants would also be advantageous in order to get sufficient statistical power to make a conclusion about the bias development with time.

Unless the factory calibration of FL can be improved to eliminate the bias errors we observe, voluntary user-supplied bias correction would be a desirable functionality in FL for patients and researchers alike. This correction could be done as in normal CGM systems, e.g., using one or two SMBG measurements per day (but in the case of FL, they could be voluntary). Such measurements should ideally be taken at periods of low glucose variability to minimize the influence of lag on the bias correction. The development of the bias over the 14-day life of the sensor is not known, so a bias correction of the whole period based on only one or two SMBG measurements from day 1 is not necessarily the right thing to do. Most studies of FL performance report that MARDs improve over time, suggesting that biases may be decreasing over time. This seems to be the case also in our data, at least for the group of participants that had a large negative bias in day 1. If so, a bias correction based only on data from the first day of use could be detrimental to the performance of the sensor in the subsequent days, as it could give an over-correction. The best way to include a voluntary bias correction in real-time use of the device is not clear. The methods that have been used by CGM systems for this purpose [9] are likely applicable, however these require daily calibration measurements. Researchers planning to use FL in studies should consider adding mechanisms for post-study bias correction of FL data in the study designs, for instance by including sufficient reference measurements to evaluate the bias at the beginning, middle and end of the study.

Bias is emphasized in this work, since it is potentially easily corrected for in real time. However, lag is also a significant reason for high MARDs, as seen in Figure 5. The individual MARDs remaining after bias correction are well correlated with the estimated lags. Correction of the lag gives a further improvement in overall MARD, however the bias contribution to MARD dominates, and lag correction only has an effect on MARD when the bias is also removed. Correction of bias has been commonplace in CGMs, through their daily calibration against SMBG measurements. Real-time correction of lag is possible in theory, but only if the lag is a pure time constant that does not change significantly over time. If instead a pure time delay is present, causality prohibits real-time correction of the lag. Practically feasible correction of a time constant requires quite noise free FGM data to avoid introducing new error by the lag correction. Lag correction also needs knowledge of the lag per participant and sensor, which requires combining FGM/CGM data with SMBG data with a similar sampling frequency as we used in day 1 of our study. This is practically unfeasible and not acceptable to most users, especially if it must be repeated for every new sensor.

Finally, it should be acknowledged that most would consider CGMs with MARD values greater than 20% unsuited for use by patients to guide them on their actual glucose levels, and even less so in assisting on deciding insulin doses. Consequently, both patients and health care personnel should be informed that some of the FL sensors have this limitation. Large positive biases as those experienced by three of our participants are particularly problematic, since they represent a risk of failing to detect hypoglycemia or impending hypoglycemia. Since our analysis is based mainly on data from day 1 of the sensor wear, which is known to be less accurate than data from subsequent days of wear, in a sense it represents a worst case analysis of the Freestyle Libre performance. It is still important for users and caretakers to know in what way the sensor could be inaccurate also in the first day of wear, since the FL presents glucose estimates to the user 60 min after sensor insertion without any warning to the user that the results are more inaccurate in day 1.

## 5. Conclusions

The observed overall MARD between SMBG and FL in the first day of use was 12.3%. However, MARDs in individual participants ranged from 4% to 25%. Many of the high individual MARD cases are caused primarily by bias. Lag and other effects are also present. The biases seem to persist beyond day 1 of wearing the FL. The FL is factory calibrated, and manual bias correction by the user is not possible. Our data indicate that the manufacturer and some patients could benefit from introducing a *voluntary* calibration mechanism in the FL, which could result in an improved MARD for some users. This calibration should likely be based on the same kind of principles as those in conventional CGM systems, i.e., using SMBG measurements from periods of low glycemic variation. Researchers using the FL may gain from designing their studies to allow for an external bias correction. Patients and health care personnel should be informed about the risks of measurement error in FGM devices, and how these errors may manifest themselves on an individual level. Further research is needed to determine if the bias follows the sensor or the patient, to investigate in more detail how the biases and lags evolve over the lifetime of the sensor, and possibly to exploit methods for detection and mitigation of biases and lags.

## Figures and Tables

**Table 1:** Participant demographics, mean (range).

Sex	14 female, 25 male
Age	42 (18–72) years
Duration of diabetes	23 (3–45) years
Body Mass Index (BMI)	27 (21–38) kg/$m^{2}$

**Table 2:** Influence on overall MARD of different signal processing of day 1 data to correct for bias and lag.

Signal Processing	Symbol	MARD (%)	MAD (mmol/L)	PEG zone A/A+B (%)
None (raw FGM scans vs SMBG)	$y_{FGM}$	12.3	1.0	81.7/100
Bias corrected, multipoint	$y_{FGM,Bcorr}$	9.2	0.8	91.2/100
Bias corrected, 1-point	$y_{FGM,Bcorr1p}$	11.4	0.9	83.0/100
Bias corrected, 2-point	$y_{FGM,Bcorr2p}$	9.7	0.7	87.7/100
Bias and lag corrected, multipoint	$y_{FGM,BLcorr}$	6.6	0.5	97/100
Only lag corrected, multipoint	$y_{FGM,Lcorr}$	11.7	0.9	81.5/100
Only smoothed, multipoint	$y_{FGM,smoothed}$	11.9	1.0	82.2/100

**Table 3:** Group summaries from bias persistence analysis. Mean: Mean of the errors [mmol/L]. SD: Standard deviation of the errors [mmol/L]. N: number of paired points. †: Group mean in days 2–7 is significantly different from the group mean in day 1. *: Group mean is significantly different from the unbiased group mean (days 2–7).

Group	Number of Participants	Mean ± SD (N) Day 1	Mean ± SD (N) Days 2–7
Positive bias	3	1.28±1.03 (96)	1.35±1.44 (35) *
Unbiased	28	0.09±1.06 (755)	0.14±1.00 (270)
Negative bias	8	−1.45±0.87 (202)	−0.74±0.73 (51) †*

**Figure A1:**
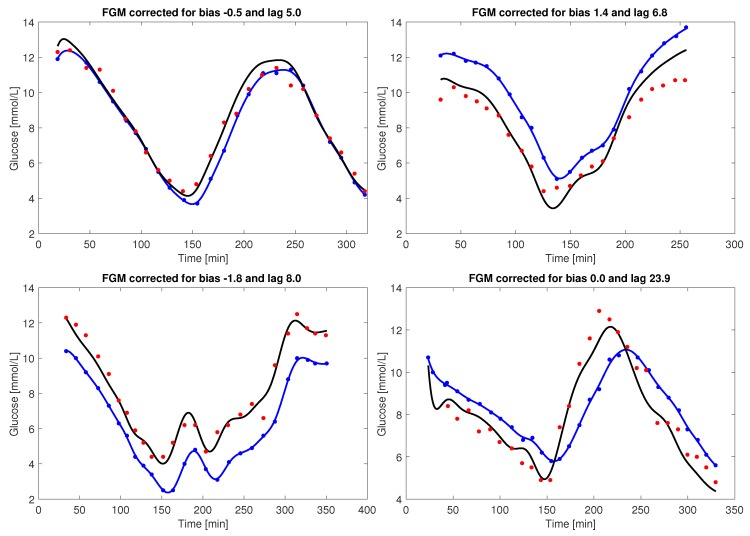
Bias and lag correction of the same data as shown in Figure 1. Here, only the FGM (**blue**) points, a bias and a lag are used as inputs to the smoother, and the black line shows the corrected signal. Red points are SMBG measurements. We see that the bias and lag correction brings the corrected FGM signal close to the SMBG measurements in all cases seen here.

## Supplementary Materials

Supplementary materials can be accessed at http://www.mdpi.com/2079-6374/8/4/93/s1.

## Abbreviations

The following abbreviations are used in this manuscript:

MARD	Mean Absolute Relative Difference
PARD	Precision Absolute Relative Difference
FGM	Flash Glucose Monitor
CGM	Continuous Glucose Monitor
SMBG	Self Monitoring of Blood Glucose
NFC	Near Field Communication
FL	Freestyle Libre
DG5	DexCom G5
CI	Confidence Interval

## Appendix A. Kalman Smoothing to Correct for Bias and Lag

Given an FGM signal with a known bias $b_{FGM}$ and a lag $\tau_{i}$, we would like to compute a signal that is corrected for both effects. We use the model of glucose dynamics given as Model 2 in the paper by Staal et al. [12], and augment it with the plasma-interstitial model given by Equations (3) and (4)), where $\tau_{i}$ and $b_{FGM}$ are now fixed quantities. The overall model becomes:

(A1) $$\begin{array}{cc} \dot{G_{p}} & =C_{r} \end{array}$$

(A2) $$\begin{array}{cc} \dot{C_{c}} & =-\frac{1}{T_{d}}C_{c}+v_{c}\left(t\right) \end{array}$$

(A3) $$\begin{array}{cc} \dot{C_{r}} & =\frac{1}{T_{d}}\left(C_{c}-C_{r}\right) \end{array}$$

(A4) $$\begin{array}{cc} {\dot{G}}_{i} & =\frac{1}{\tau_{i}}\left(G_{p}-G_{i}\right) \end{array}$$

where $T_{d}$ is a parameter describing the flow between the internal compartments $C_{c}$ and $C_{r}$, set to 10 min. $v_{c}\left(t\right)$ is process noise affecting state $C_{c}$. For more information about the model, and choice of parameters, process and measurement noises, see [12].

This system is linear, and can be put on the form $\dot{x}=A\left(\tau_{i}\right)x$, and a discretized version of the system is $x_{k+1}=\Phi x_{k}$, where $\Phi =e^{A(\tau_{i})\Delta t}$. We used Δt=0.5 min.

The Kalman filter equations are [15]:

(A5) $${\bar{x}}_{k}=\Phi_{k-1}{\hat{x}}_{k-1}$$

(A6) $${\bar{P}}_{k}=\Phi {\hat{P}}_{k-1}\Phi^{T}+Q$$

(A7) $$K_{k}={\bar{P}}_{k}H^{T}{\left(H{\bar{P}}_{k}H^{T}+R_{k}\right)}^{-1}$$

(A8) $${\hat{x}}_{k}=K_{k}\left(y_{k}-b_{FGM}-H_{k}{\bar{x}}_{k}\right)$$

(A9) $${\hat{P}}_{k}=\left(I-K_{k}H\right){\bar{P}}_{k}$$

here *H* is a measurement matrix, *Q* is the process noise covariance matrix, and *R* is the measurement matrix. *P* matrices are state covariance matrices propagated by the filter. We used $H=[\begin{array}{cccc} 0 & 0 & 0 & 1 \end{array}]$, and we subtracted the bias $b_{FGM}$ from each measurement as part of the Kalman filter measurement update Equation (A8). The *Q* matrix we used was all zero, except for the diagonal element corresponding to $C_{c}$, this was set to 0.025. *R* (scalar in our case) is set for each measurement based on ISO 15197 limits, as described in [12].

The Rauch-Tung-Striebel (RTS) algorithm [14] makes use of stored data from the Kalman filter forward pass, namely the sequences of a priori and a posteriori state estimates ${\bar{x}}_{k}$, ${\hat{x}}_{k}$ and state covariance matrices ${\bar{P}}_{k}$ and ${\hat{P}}_{k}$. These are input to a backward pass that computes the smoothed estimates ${\hat{x}}_{k}^{s}$ and ${\hat{P}}_{k}^{s}$ as follows [15]:

(A10) $$C_{k}={\hat{P}}_{k}\Phi {\bar{P}}_{k+1}^{-1}$$

(A11) $${\hat{x}}_{k}^{s}={\hat{x}}_{k}+C_{k}\left({\hat{x}}_{k+1}^{s}-{\bar{x}}_{k+1}\right)$$

(A12) $${\hat{P}}_{k}^{s}={\hat{P}}_{k}+C_{k}\left({\hat{P}}_{k+1}^{s}-{\bar{P}}_{k+1}\right)C_{k}^{T}$$

After smoothing is completed, the bias and lag corrected signal is available as the first element in the ${\hat{x}}^{s}$ signal, corresponding to the $G_{p}$ state. Examples of the result of the state estimation Kalman smoother is given in Figure A1.

The smoother does not introduce any additional delay in the smoothed signal, by virtue of its forward-backward nature.

The Matlab code of the Kalman smoother for use in glucose applications is publicly available, see [12].

## Appendix B. MARD Analysis of Day 2–7 Data Using Different Bias Correction Approaches

**Table A1:** Influence on overall MARD of different signal processing for days 2–7, using biases and lags estimated from day 1 data.

Signal Processing	Symbol	MARD (%)	MAD (mmol/L)	PEG zone A/A+B (%)
None (FGM historic data vs SMBG)	$y_{FGM}$	11.3	0.8	87.3/99.2
Bias corrected, multipoint	$y_{FGM,Bcorr}$	12.5	0.8	89.0/97.7
Bias corrected, 1-point	$y_{FGM,Bcorr1p}$	13.8	0.9	82.4/99.7
Bias corrected, 2-point	$y_{FGM,Bcorr2p}$	12.2	0.8	89.2/99.7
Bias and lag corrected, multipoint	$y_{FGM,BLcorr}$	12.7	0.8	86.7/97.5
